# Vanillin: A Promising Biosourced Building Block for the Preparation of Various Heterocycles

**DOI:** 10.3389/fchem.2022.949355

**Published:** 2022-07-07

**Authors:** David Gendron

**Affiliations:** Kemitek, Cégep de Thetford, Thetford Mines, QC, Canada

**Keywords:** vanillin, biosourced, heterocycles, green chemistry, synthesis, aromatic ring

## Abstract

The preparation of heterocyclic compounds often involves the use of petroleum-based or non-renewable sources. Considering the actual societal and environmental awareness towards sustainable chemistry, new and green sources of organic carbon are sought. In this regard, vanillin is a molecular building block that can be obtained from the depolymerization of lignin. Due to its different functional groups (hydroxyl, aldehyde, and methoxy) vanillin can undergo a variety of reactions leading to various heterocycles such as pyrimidines, quinoxalines, imidazoles or thiazoles to name a few. This mini-review will focus on the preparation of accessible heterocycles building blocks from the vanillin moiety in regard to the medicinal, pharmaceutical, and material fields.

## Introduction

Nowadays, the preparation of heterocyclic compounds mostly starts with a petroleum-based sources as they are readily available and for some cases, inexpensives. However, due to an ever-increased demand, a shortage of these non-renewable resources is to be anticipated together with an augmentation of their cost. Therefore, it is of a paramount importance that other alternatives are sought. In recent years, there is a societal and economical awareness towards a more sustainable chemistry using renewable sources of organic carbon. In this regard, the forest biomass is considered a promising source, as it is renewable, abundant, and principally composed of carbohydrates (cellulose and hemicellulose) as well as aromatic derivatives (lignin and tannin) (Zhu et al., 2016). More specifically, several chemical compounds can be obtained such as organic acids (glutamic, lactic, and levulinic), alcohols (ethanol, glycerol, and sorbitol), and phenols (vanillin, catechol, and eugenol) (Isikgor et al., 2015).

A compound of interest that can be obtained from the depolymerization of lignin, is vanillin. As a matter of fact, vanillin can be obtained from lignin by three different processes: 1) the sulfite process: wood is subjected to a sulfite pulping process (140–170°C) affording the lignosulfonate-rich sulfite liquor. Then removal of the sugars by ultrafiltration and oxidation of the lignosulphonates yields vanillin in <10% (Bjørsvik et al., 1999); 2) the kraft process: wood fibers are digested in a mixture of sodium hydroxide and sodium sulfite at 170°C. During this depolymerization process, the *α* and *β* aryl ether bonds are cleaved in both phenolic and non-phenolic moieties (Lora et al., 2008); and 3) the soda process: in this case the fibrous feedstock is decomposed with an aqueous solution of NaOH. The process is carried out at 160°C and as for the kraft process, cleavage of the lignin-carbohydrate linkage takes place affording phenolic and non-phenolic units (Lora et al., 2008). Vanillin is typically obtained in higher yields from hardwood lignin (30%–50%) than softwood lignin (17%–28%) (Tarabanko et al., 2017). It is also important to point out that industrially, only lignin from the sulfite pulping process is employed to afford vanillin (Fache et al., 2016). At the moment, biosourced vanillin is produced from the company Borregaard from the Norway spruce tree (soft wood). Interestingly, vanillin can be obtained from plant biomass such as wheat straw by alkaline oxidation (Na_2_CO_3_, O_2_, 11–21 bar, 195°C) although in low yield (<0.1%) (Klinke et al., 2002). In terms of sourcing, vanillin is produced from oil (85%), forest biomass (15%) and from the vanilla orchid pods (<1%) (Fache et al., 2016). In the latter, vanillin is obtained from the enzymatic hydrolyzation of vanillin glucoside found in the beans (Hocking et al., 1997).

**FIGURE 1:**
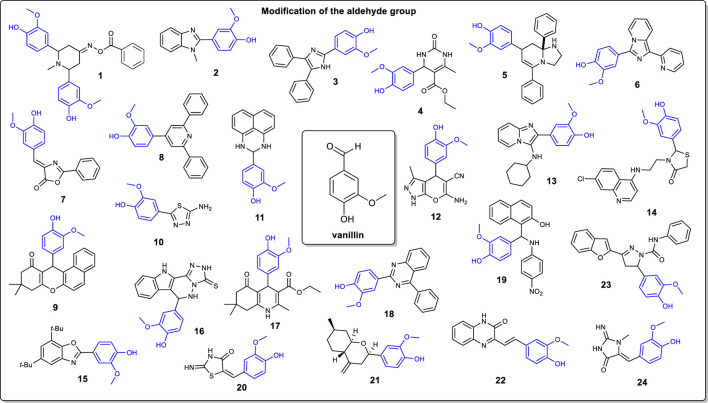
Examples of the modification of the vanillin moiety through the aldehyde functional group.

**FIGURE 2:**
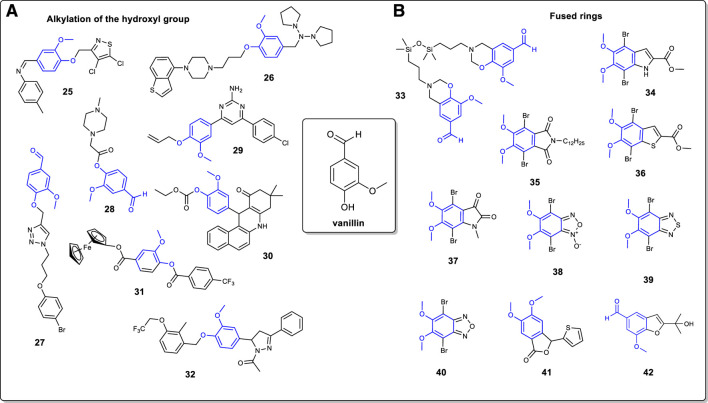
**(A)**: Alkylation of the vanillin moiety through the hydroxyl functional group; **(B)**: Vanillin-based heterocyclic fused rings.

Vanillin possesses a unique aromatic structure and three functional groups (methoxy, aldehyde, and hydroxyl, see Figure 1) that can be chemically modified. At the moment, vanillin is principally used as an additive in food (bakeries and drinks), in perfumes and in pharmaceutical compounds. This mini-review discuss the modification of the vanillin moiety into heterocyclic compounds. Three main transformations are reviewed: 1) the modification of the aldehyde group, 2) the alkylation of the hydroxyl group, and 3) the formation of fused rings. At last, the oxidative ring opening reactions of the vanillin molecule is discussed. The molecules shown here (Figures 1, 2) found their utility in a variety of domains ranging from medicinal chemistry to materials science.

## Modification of the Aldehyde Group

As stated earlier, the main modification of the vanillin moiety is through the aldehyde functional group. Indeed, a plethora of heterocycles can be synthesized. However, most of the structures reported are nitrogen-based five-and six-membered rings. A first example is the synthesis of the piperidin-4-one oxime ester 1 as shown by Harini and coworkers (Harini et al., 2012). In their work, vanillin is subjected to a condensation reaction (Mannich reaction) in presence of acetone and ammonium acetate (2:1:1 ratio respectively). Then, the latter obtained piperidi-4-one intermediate is methylated with methyl iodide followed by treatment with hydroxylamine hydrochloride to afford the corresponding oxime. Esterification of the oxime with *t*-BuOK, and reaction with benzoyl chloride gave compound 1. Also, the preparation of aryl benzimidazoles is a common modification of the vanillin molecule. Jin and Chakrabarty both reported the preparation of compound 2, but with two different approaches (Chakrabarty et al., 2007; Jin et al., 2014). Jin and coworkers proposed the conversion of vanillin directly into benzimidazole 2 with 90% yield using *o*-phenylenediamine and FeCl_3_.6H_2_O under ball milling conditions. Alternatively, Chakrabarty and coworkers prepared compound 2 by reacting vanillin with silicotungstic acid and *N*-methyl-*o*-phenylenediamine in 60% yield.

Recently, different ionic liquids were used as catalysts and reaction mediums for the preparation of several vanillin-based derivatives. Ahmed and coworkers have reported the synthesis of trisubstituted imidazole from vanillin using an ionic liquid as the catalyst (Ahmed et al., 2021). In this example, vanillin is converted into imidazole derivative 3 in presence of benzil and acidic ionic liquid ([{(IMC)-4-OMBH}BIM][HSO_4_]_3_) which was prepared in-house. Alvim and coworkers demonstrated the use of a Biginelli multicomponent reaction for the preparation of heterocycle 4 (Alvim et al., 2018). Similarly to Ahmed et al. (2021) they reported the use of a unique ionic liquid (MSI.TRIP) acting as the catalyst of the reaction. In this case, vanillin is reacted with ethyl-3-oxobutanoate, urea, and MSI.TRIP to generate derivative 4. Interestingly, there are no solvent required and there are no reactant’s specific addition order needed to carry out the reaction. In the same vein, ionic liquid MSI_3_PW was used as a catalyst for the preparation of hexahydroimidazo [1,2-*a*]pyridine compound **5** (Alvim et al., 2018). Vanillin (1 Eq) is reacted with ethylenediamine (1 Eq.) and benzaldehyde (2 Eq.) in ionic liquid BMI.BF_4_ as solvent and MSI_3_PW as the catalyst. The multicomponent synthesis of compound 5 is completed in 55% yield. 1-pyridylimidazo [1,5-*a*] pyridines compound 6 is prepared from reaction between 1,2-dipyridylketone and vanillin using 1-butylimidazolium tetrafluoroborate ([Hbim]BF_4_) as a promoter for the heterocyclization reaction (Siddiqui et al., 2006). The reaction is quite versatile on the choice of aldehyde substrates and the ionic liquid can be recycled. Similarly, ionic liquid [Et_3_NH][HSO_4_] has been used for the stereoselective preparation of 4-arylidene-2-phenyl-5(4*H*)-oxazolone 7 [(Parveen et al., 2015). In this example, vanillin is reacted with hippuric acid and triethylammonium sulfate to afford compound 7 in 94% yield. Interestingly, performing the reaction in acetic anhydride led to a lower yield 72%] and longer reaction time.

On another note, Bai and coworkers discussed an interesting way to access [3 + 2 + 1] pyridine skeletons in one step (Bai et al., 2021). Starting from vanillin, a cascade carbopalladation-cyclization is carried out in presence of phenylboronic acid, palladium acetate (catalyst), 6-methyl-2,2′-bipyridine (bidentate ligand), and 1-fluoro-2,4,6-trimethylpyridin-1-ium trifluoromethanesulfonate as the oxidant, affording compound 8 in 71% yield. In terms of involving renewable reactants for the preparation of vanillin-based heterocycle, Ma et al. (2018) have proposed the use of a carbonized xylan-type hemicellulose as a catalyst for a three-component condensation. In this example, vanillin is reacted with 2-naphthol and dimedone in presence of CXH-SO_3_H to afford benzoxanthene compound 9. Another interesting heterocycle that can be obtained from vanillin is the 1,3,4-thiazole moiety (Rebolledo et al., 2013). The first step is the acetylation of the hydroxyl group with anhydride acetic followed by the formation of a thiosemicarbazone intermediate in presence of thiosemicarbazide. Then, a oxidative cyclization of the previous intermediate is carried out with iron (III) chloride to afford 2-amino-1,3,4-thiadiazole derivative 10. In a different approach, Varsha and coworkers reported the preparation and crystallographic data of a heterocyclic pyrimidine (Varsha et al., 2010). Compound 11 is obtained in one step from the reaction of vanillin and 1,8-diaminonahpthalene under reflux. Crystal structure of compound 11 showed two independently twisted crystal structures.

The synthesis of pyrano [2,3-c] pyrazole derivative 12 was reported by Tekale and coworkers (Tekale et al., 2013). Their study proposes a greener synthetic approach involving water as the reaction medium and ZnO nanoparticles as the catalyst that can be recycled. The reaction proceeds according to the following mechanistic steps: 1) formation of the pyrazolone ring by reaction between ethyl acetoacetate and hydrazine, 2) Knoevenagel condensation between vanillin and malononitrile, 3) Michael addition of the pyrazolone with the obtained 2-(4-hydroxy-3-methoxybenzylidene) malononitrile followed by 4) cyclization and tautomerization, generating compound 12.

Several studies report the one-pot preparation of vanillin-based heterocycles. For instance, Luo and coworkers proposed the synthesis of a *α*-ketoamide for antiviral and antibacterial activities using vanillin as a lead and starting material (Luo et al., 2020). Compound 13 is prepared in a one-pot *β*-cyclodextrin-SO_3_H-based catalytic process. More precisely, vanillin is mixed with 2-amino-pyridine, isocyanocyclohexane and the catalyst just mentioned affording targeted compound 13. Ruiz and coworkers reported the synthesis of a hybrid heterocyclic compound based on chloroquine and thiazolidinone moieties (Ruiz et al., 2011). The first step is the introduction of a *α,ω*-diaminoalkane on the 4,7-chloroquinoline (DCQ) ring. Then, the latter is reacted with vanillin and *α*-mercaptoacetic acid in a one-pot three components to afford targeted compound 14. On another note, Sharghi and coworkers reported a one-pot multicomponent synthesis of a vanillin-based benzoxazole (Sharghi et al., 2020). To carry out the transformation, vanillin was reacted with 3,5-di-tert-butylbenzene-1,2-diol, ammonium acetate and an iron (III)-Salen complex as catalyst generating compound 15. Boraei and coworkers have reported the synthesis of a heterocycle containing three fused rings: indole, 1,2,4-triazole and quinoxaline (Boraei et al., 2020). The preparation is carried out in a one-step multicomponent involving 4-amino-5-(1H-indol-2-yl)-2,4-dihydro-3H-1,2,4-triazole-3-thione with vanillin in concentrated HCl, affording compound 16 and yielding exclusively the targeted compound. Fatma and coworkers proposed a one-pot synthesis of polyhydroquinoline *via* a Hantzsch reaction (Fatma et al., 2014). In this example, vanillin was reacted with ethyl 3-oxobutanoate, dimedone, ammonium acetate, and thiamine hydrochloride as the catalyst. Condensation product 17 is obtained in 89% yield. Lastly, vanillin can be converted into a 2,4-disubstituted quinazoline in a one-pot reaction under relatively mild conditions (Bose et al., 2020). Interestingly, in this process, 2,3-dichloro-5,6-dicyanobenzoquinone (DDQ) is used as a catalyst to convert vanillin in presence of *o*-aminobenzophenone into compound 18. The reaction proceeded free of self-condensation of carbonyl compounds. Mechanistically, the amine does react with the aldehyde group of the vanillin moiety, affording a Schiff’s base intermediate. Then, a cyclization occurs followed by a DDQ mediated aromatization (*via* oxidation) to generate quinozaline 18.

Tannic acid was used as the catalyst for the preparation of aminoalkylnaphthol derivative 19 from vanillin (Deepam et al., 2017). Two different approaches were compared: 1) thermal heating (oil bath) and 2) microwave irradiation. In both methods, vanillin was reacted with 2-naphthtol, 4-nitroaniline and tannic acid as the catalyst to give compound 19 with similar yields (90%). Compound 20 was also obtained under microwave irradiation (MW) conditions (Awate et al., 2020). Vanillin was reacted directly with thiourea and 2-chloroacetic acid under MW to give (E)-5-(4-hydroxy-3-methoxybenzylidene)-2-iminothiazolidin-4-one compound 20.

Octahydro-2*H*-chromen-4-ol is a type of heterocycle that can be obtained from vanillin (Timofeeva et al., 2015). The synthesis proceeded via an acid catalyzed Prins cyclization reaction with isopulegol, vanillin and montmorillonites as the catalyst, affording compound 21. As previously discussed, vanillin was reacted with malononitrile towards the preparation of quinoxaline units. Mahajan et al. reported the synthesis of a styrylquinoxalin-2 (1*H*)-ones (Mahajan et al., 2020). In this instance, vanillin was mixed with malononitrile and 3-methylquinoxalin-2 (1H)-one and subjected to microwave irradiation affording compound 22. Benzofuran 1,3,5-substituted pyrazole compound 23 was prepared in four steps from vanillin (Rangaswamy et al., 2012). First, vanillin was reacted with 2-acetyl benzofuran in presence of a ZrCl_4_ as the catalyst to afford (E)-1-(benzofuran-2-yl)-3-(4-hydroxy-3-methoxyphenyl) prop-2-en-1-one intermediate. The latter was subjected to a Claisen-Schmidt cyclocondensation with hydrazine hydrate followed by treatment with phosgene and triethylamine furnished 3-(benzofuran-2-yl)-5-(4-hydroxy-3-methoxyphenyl)-4,5-dihydro-1H-pyrazole-1-carbonyl chloride intermediate. The final step was the addition of aniline to generate the carboxamide compound 23. At last, 5-(4-hydroxy-3-methoxybenzal) creatinine compound 24 was prepared by simply heating a mixture of vanillin and creatinine at 160°C in 81% yield (Fowler et al., 1992).

## Alkylation of the Hydroxyl Group

Although less frequent, it is possible to alkylate the hydroxyl group at the 4-position on the vanillin ring. In this regard, a common way is to convert the hydroxyl into a methoxy using a methylating agent such a methyl iodide or dimethyl sulfate (Gavara et al., 2010; Selvaraju et al., 2015). However, more complex heterocyclic structure can also be prepared (Figure 2). Kletskov and coworkers have reported the preparation of an isothiazole derivative (Kletskov et al., 2017). Vanillin is alkylated in presence of 4,5-dichloro-3-chlormethylisothiazole to generate the corresponding ether which is then reacted with *p*-toluidine to afford the azomethine 25. On another note, Gao and coworkers discussed the preparation of a piperazine heterocycle incorporating a vanillin moiety 26 (Gao et al., 2021). First, vanillin was converted into an amine intermediate by reaction with pyrrolidine and triacetoxyborohydride. The latter was then alkylated with a benzothiophenylpiperazine chloride derivative to afford compound 26. As reported by Hussain and coworkers, it is possible to use the vanillin moiety in 1,3-dipolar cycloaddition (“click” chemistry) for the preparation of 1,4-disubstituted 1,2,3-triazoles (Hussain et al., 2019). In this example, vanillin is alkylated with propargyl bromide followed by a 1,3-dipolar cycloaddition using Cu (I) as catalyst and a 3-azidopropoxy arene to afford compound 27. Cu (I) was generated *in situ* by the reduction of CuSO_4_. 5H_2_O with sodium ascorbate. In the same vein, Ashraf and coworkers have also reported the synthesis of a piperazine-vanillin analog (Ashraf et al., 2015). Using chloroacetyl chloride as reactant, vanillin was converted to its chloroacetyl intermediate. The latter was then reacted with methyl-piperazine to afford compound 28 in 81% yield.

It was shown that the preparation of a vanillin allylated pyrimidine derivative can be carried out in four steps (Sharma et al., 2014). In this instance, vanillin is first reacted with allyl bromide followed by reaction with the appropriated acetophenone to generate the (E)-3-(4-(allyloxy)-3-methoxyphenyl)-1-(4-chlorophenyl) prop-2-en-1-one chalcone intermediate. Then, reaction with guanidine led to compound 29. Vanillin can be modified into benzo [*a*] acridinone 30 through a Schiff base intermediate (Dikusar et al., 2007). Vanillin is sequentially acetylated with ethyl chloroformate, converted into its corresponding Schiff base with naphthalen-1-amine and condensed with dimedone to afford compound 30.

Vanillin has been utilized as a precursor to the preparation of ferrocenyl 4-trifluoromethylbenzoate 31, a potential antitumor compound (Denisov et al., 2016). The first step is the reaction of vanillin with *p*-trifluorobenzaldehyde and DABCO under microwave irradiation condition. The compound thus obtain was reacted with ferrocene boronic acid to give compound 31 in a low yield of 15%. Finally, Patel and coworkers reported the preparation of a vanillin-based pyrazoline antibacterial agent (Patel et al., 2014). Vanillin was subjected to 2-(chloromethyl)-3-methyl-4-(2,2,2-trifluoroethoxy) pyridine hydrochloride to form the corresponding ether. The latter was then subjected to a Claisen-Schmidt condensation reaction with acetophenone, followed by reaction with hydrazine hydrate to furnish pyrazoline compound 32.

## Fused Rings

The vanillin molecule can be modified to form several aromatic and non-aromatic fused rings (Figure 2). The transformation occurs mostly at the aldehyde site but is also possible to involve the hydroxyl group. For instance, Sharma and coworkers reported the preparation of a several benzoxazines units. In their work, vanillin undergoes a Mannich like condensation with 1,3-bis (3-aminopropyl) tetramethyldisiloxane and paraformaldehyde to form the bio-based benzoxazine 33 (Sharma et al., 2020). To cope with the high viscosity of the reaction, ethanol was chosen as an appropriate reaction medium. Several groups have reported the used of the vanillin moiety to prepare conjugated fused ring monomers for applications in the field of organic electronics. Both Huleatt and Selvaraju reported the synthesis of indole derivative 34, but with a different synthetic approach (Huleatt et al., 2008; Selvaraju et al., 2015). In the first case, vanillin is reduced to dihydroxybenzaldehyde and then sequentially brominated, methylated at the 5,6 positions, and condensed with methyl azidoacetate to form a dibromoazidocinnamate intermediate (Huleatt et al., 2008). Finally, the latter is cyclized to give compound 34 in low yield as a side product. Alternatively, it was possible to carry out the methylation of vanillin followed by nitration and bromination steps to give 2,5-dibromo-3,4-dimethoxy-6-nitrobenzaldehyde (Selvaraju et al., 2015). Then, it was converted to an olefin intermediate with the use of methyl bromoacetate followed by a Cadogan ring closure reaction to afford compound 34 in an overall yield of 40% starting from vanillin. Heteroaromatic compounds 35–40 were obtained from a renewable source of vanillin (Boivin et al., 2021). The phthalimide derivative 35 was synthesized in five steps. Starting from methylated vanillin, an iodination reaction gave 2-iodo-4,5-dimethoxybenzaldehyde which was converted to 4,5-dimethoxyphthalonitrile over two steps. Then, the latter is reacted with NaOH and then sequentially reacted with acetic anhydride, dodecylamine and *N*-bromosuccinimide (NBS) to afford compound 35. Concerning benzothiophene derivative 36, it was obtained from a similar synthetic pathway as just described for compound 34. However, the 2,5-dibromo-3,4-dimethoxy-6-nitrobenzaldehyde intermediate was reacted with methyl thioglycolate to give directly compound 36 in low yield (19%). Interestingly, the isatin derivative 37 can be prepared from compound 34. In this example, compound 34 is first saponified followed by decarboxylation of the carboxylic acid functional group. The indole intermediate thus obtained is methylated with dimethyl sulfate and converted to compound 37 by an oxidation reaction involving I_2_O_5_.

Benzofuroxan compound 38 was prepared from vanillin in six steps. The key step is the one pot transformation of the amide and nitro groups through a Hoffman rearrangement of the 4,5-dimethoxy-2-nitrobenzamide intermediate to convert the amide into an amide, thus generating the furoxan moiety. Finally, compounds 39 and 40 are obtained from compound 38. The benzothidiazole compound 39 was prepared straight from compound 38 by a reduction of the furoxan ring with tin chloride into an unstable intermediate 4,5-dimethoxybenzene-1,2-diamine which was subjected to thionyl chloride to generate the benzothiadiazole ring. Bromination of the latter afforded compound 39. Reaction of the benzofuroxan unit 38 with triphenylphosphine followed by a bromination reaction (Br_2_, AcOH) leads to benzofurazan compound 40 in two steps only.

Vanillin can also be used for the preparation of isomeric phthalides (Costa et al., 1988). Vanillin is first methylated using dimethylsulfate and then converted to a dimethylketal intermediate [4-(dimethoxymethyl)-1,2-dimethoxybenzene] in presence of ammonium nitrate. Then, a regioselective lithiation reaction at the 2-position generates a dihydroisobenzofuran intermediate which was oxidized with pyridinium chlorochromate to furnish phthalide compound 41. At last, it is possible to access benzofuran rings *via* a Sonogashira coupling reaction using vanillin as the starting compound (Succaw et al., 2009). In the latter, vanillin is first converted to 5-iodovanillin followed by reaction with 2-methyl-3-butyne-2-ol using Pd (OAc)_2_, CuI, a phosphine ligand and *N*-methylmorpholine to afford compound 42 *via* the ortho phenol substituent.

## Oxidative ring opening

The oxidative ring opening of phenol-like compounds is a major industrial process to access several useful building blocks. For instance, vanillin can be oxidized by gaseous ozone and hydroxyl radicals to furnish compounds such as maleic acid, glyoxylic acid or pent-2-enedioic acid to name a few (Rana et al., 2020). Another approach is the cleavage of the vanillin C-C and C-O of bonds using microwaves and copper oxide as catalyst generating organic diacids (i.e., fumaric acid, maleic acid, malic acid, and succinic acid) (Qu et al., 2020). Alternatively, microorganisms can also catalyze the ring cleavage of vanillin. For instance, Artaud and coworkers reported the oxidative conversion of the vanillin moiety into a diene chromophore using *Pseudomonas cepacia* as catalyst (Artaud et al., 1995). Similarly, the *Lentinus edodes* enzyme was able to perform the aromatic ring oxidation of vanillin-like compounds (Crestini et al., 1995).

## Summary and Outlook

As discussed in this mini-review, vanillin can be employed as a core building block for the preparation of complex heterocycles. In particular, the synthesis of various five-and six-membered rings incorporating nitrogen, oxygen or sulphur atoms was reviewed. Vanillin can also be biosourced, and thus, represents a promising alternative to similar core molecules obtained from non-renewable resources. Consequently, the exploration of other environmentally sustainable molecules must be pursued to enable a greener chemistry, one step at the time.
